# Epigenetic regulation of the oxytocin receptor is associated with neural response during selective social attention

**DOI:** 10.1038/s41398-018-0159-x

**Published:** 2018-06-15

**Authors:** Meghan H. Puglia, Jessica J. Connelly, James P. Morris

**Affiliations:** Department of Psychology, University of Virginia, Charlottesville, VA USA

## Abstract

Aberrant attentional biases to social stimuli have been implicated in a number of disorders including autism and social anxiety disorder. Oxytocin, a naturally-occurring mammalian hormone and neuromodulator involved in regulating social behavior, has been proposed to impact basic biological systems that facilitate the detection of and orientation to social information. Here, we investigate a role for naturally-occurring variability in the endogenous oxytocinergic system in regulating neural response during attention to social information. Participants performed a selective social attention task while undergoing fMRI, provided a blood sample for epigenetic analysis, and completed self-report measures of social functioning. We find that a functional epigenetic modification to the oxytocin receptor, *OXTR* methylation, is associated with increased neural response within and decreased functional coupling between regions of the salience and attentional control networks during selective social attention. We also show that subclinical variability in autistic and social anxiety traits moderates this epigenetic regulation of neural response. These data offer a mechanistic explanation to a growing literature associating social behavior and disorder with epigenetic modification to *OXTR* by suggesting that *OXTR* methylation reflects a decrease in the extent to which social information automatically captures attention. We highlight the importance that treatment efficacy be considered in relation to individual differences in molecular makeup, and that future studies aimed at uncovering biomarkers of disorder carefully consider measurement at both the biological and phenotypic level.

## Introduction

Successful organisms must be able to detect and appropriately utilize important environmental cues. Stimulus detection and proper allocation of attentional resources rely upon the dynamic interplay between large-scale brain networks, specifically the salience and attentional control networks^1–3^. These separable but interrelated systems are involved in both stimulus-driven attentional orienting, and top–down, cognitive control of attentional orienting, respectively^4,5^. Together, these networks enable organisms to focus perceptual and cognitive resources on the most relevant stimulus^6,7^.

For humans, social cues are often particularly relevant and informative, and are typically considered a highly salient class of stimuli^8^. However, the extent to which social stimuli automatically capture attention varies across individuals. For example, individuals diagnosed with autism spectrum disorder (ASD) often show reduced attention to social stimuli^9–11^. To understand the neural mechanisms underlying disordered social attention, Herrington and colleagues examined social stimulus detection in the context of a distracting stimulus among individuals diagnosed with ASD^12^. Participants were asked to complete a one-back working memory task while viewing images of faces overlaid upon images of houses. Although all trials involved the simultaneous presentation of faces and houses, the key manipulation was that participants were asked to alternatively focus on either the faces or the houses when performing the one-back task. They found that individuals with ASD show increased neural response when selectively attending to faces, particularly in right dorsolateral prefrontal cortex (DLPFC), a key node in the attentional control network^2^. Conversely, typically developing control participants showed increased DLPFC activity when selectively attending to houses. The authors concluded that in neurotypical individuals, additional neural resources are required to *ignore* the social stimulus, whereas in the clinical population, attentional control mechanisms are instead required to *discern* the social stimulus in a complex visual display.

One mechanism that may contribute to such individual differences in the intrinsic salience of social information thereby driving differential recruitment of neural networks during social attention is variability within the oxytocinergic system. Oxytocin is a mammalian hormone and neuromodulator associated with the regulation of social behaviors^13,14^. In humans, the oxytocinergic system is often studied by administering synthetic oxytocin intranasally. Administration studies have shown that exogenous oxytocin has diverse and sometimes seemingly contradictory effects. For example, oxytocin administration increases prosocial behaviors such as time looking at eyes^15^, memory for faces^16^, and trust^17^, but also negative behaviors like envy^18^, anxiety^19^, and aggression^20^. The “social salience hypothesis” of oxytocin attempts to reconcile these discrepant findings by suggesting that oxytocin has a general effect on basic biological systems that facilitate detection of and orientation to social information^18,21^. This theoretical framework highlights a role for contextual (e.g., task difficulty) and individual difference (e.g., personality traits) factors that influence attention to and interpretation of social cues, and ultimately drive different downstream responses, like trust or anxiety^22,23^. For example, increasing the salience of social information through oxytocin administration might benefit individuals who are intrinsically less attentive to social cues, such as those with autism^24^, but may be detrimental to individuals who are already hypersensitive and/or predisposed to interpret social cues negatively, as is seen in social anxiety disorders^25^.

Here, we consider how naturally-occurring variability in the endogenous oxytocinergic system impacts neural response during attention to social information. The actions of oxytocin are dependent upon its receptor, which is expressed at varying levels across individuals. One factor that contributes to this variability is DNA methylation of the oxytocin receptor gene, *OXTR*. Methylation within the promoter of *OXTR* was previously shown to impact gene transcription^26,27^ and is therefore hypothesized to play a functional role in the expression of the gene such that individuals with higher *OXTR* methylation within this region are presumed to have decreased ability to use endogenous oxytocin. Gregory, Connelly et al. identified specific sites within the *OXTR* promoter that show elevated methylation in both cortex and peripheral blood in individuals with autism^27^, suggesting that this epigenetic marker may be conserved across tissue types and predictive of neural or phenotypic variability.

In the present study, we test the hypothesis that healthy individuals with increased *OXTR* methylation fail to ascribe salience to social information and therefore require the recruitment of additional attentional resources. To test this hypothesis, we employ the selective social attention paradigm previously shown to distinguish between neurotypical and ASD populations through differential activation of attentional control regions^12^, and assess methylation at a functional site identified as a reliable marker for *OXTR* expression in human cortex. In an exploratory analysis, we also consider whether subclinical autistic or socially-anxious phenotypes moderate the relationship between *OXTR* methylation and neural response during selective social attention.

## Materials and methods

### Participants

Fifty-four neurotypical Caucasians of European descent (31 males) aged 18–30 (*M* = 21.2, SD = 3.0) years participated in the present study. This target sample size was determined by calculating the average sample size of (a) previous studies^28,29^ examining the relationship between *OXTR* methylation at our target site and neural response to social stimuli (*n* = 42, *n* = 98), (b) a previous fMRI study^12^ that used this task (*n* = 31), and (c) a power analysis^30^ predicting a robust two-tailed bivariate correlation (*r* = 0.50) between methylation and neural response with power = 0.80 and *α* = 0.01 (*n* = 42). We specifically recruited a homogenous sample to avoid potential epigenotyping artifacts related to population stratification^31^ or age^32^. Methylation at the tested site is stable in the sampled age range^33^. All participants provided written informed consent for a protocol approved by the University of Virginia Institutional Review Board for Health Sciences Research and were paid $50.

### Selective social attention paradigm

**Fig. 1:**
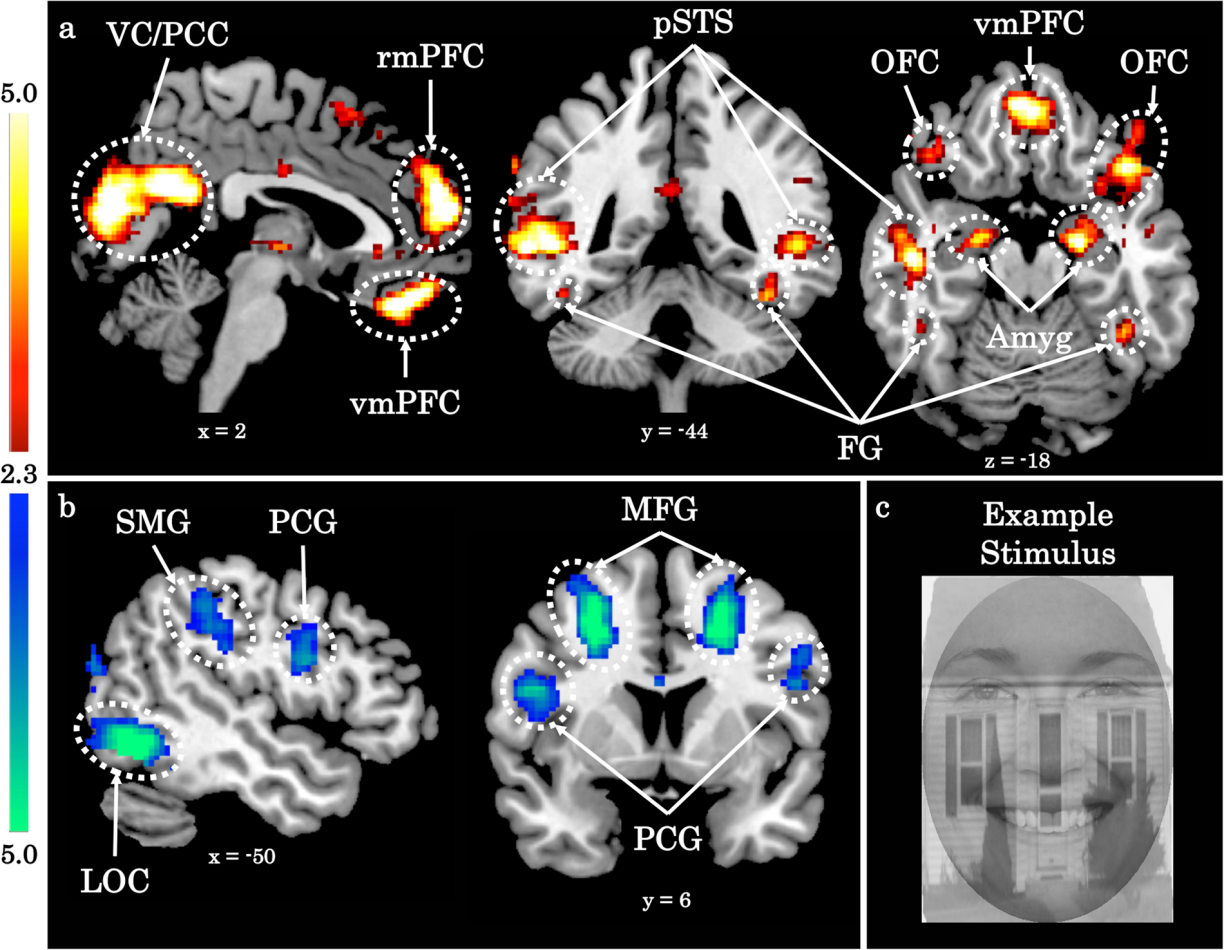
Participants activate regions of the face perception network when selectively attending to faces. **a**, **b** *Z* statistic maps of regions showing a significant main effect of the selective attention task, FDR corrected at *q* < 0.05 and *k* > 10 voxels. Images are depicted in MNI space and radiological orientation. **a** Attend Faces > Attend Houses BOLD activity. **b** Attend Faces < Attend Houses BOLD activity. **c** An example stimulus. VC visual cortex, PCC posterior cingulate cortex, rmPFC rostromedial prefrontal cortex, pSTS posterior superior temporal sulcus, OFC orbitofrontal cortex, vmPFC ventromedial prefrontal cortex, FG fusiform gyrus, Amyg amygdala, SMG supramarginal gyrus, PCG precentral gyrus, MFG medial frontal gyrus, LOC lateral occipital cortex

Participants completed a one-back selective attention task^12,34^ in which they were presented with composite images of faces and houses (see Fig. 1) while undergoing fMRI. Stimuli consisted of Caucasian faces obtained from the Chicago^35^ and Stirling/ESRC (http://pics.stir.ac.uk/) face databases, and houses photographed in neighborhoods surrounding Yale University (provided by investigators at the Center for Autism Research, Children’s Hospital of Philadelphia) and the University of Virginia. All images were converted to grayscale, matched on luminance and spatial frequency using the MATLAB SHINE toolbox^36^, and presented on a black background.

Each block began with a prompt instructing participants to attend to either the face (Attend Faces) or the house (Attend Houses) in the image when making the same/different decision. Participants completed six blocks each of the Attend Faces and Attend Houses conditions. Each block lasted 40 s and consisted of 10 images and 4–5 “same” hits. The order and pairing of face and house images were randomized for each participant. Block order was pseudorandomized for each participant such that blocks always alternated between Attend Faces and Attend Houses conditions. Stimulus presentation sequence and timing were determined using optseq2 (https://surfer.nmr.mgh.harvard.edu/optseq/). Stimuli were presented for 1800 ms with an inter-stimulus interval ranging from 200–2400 ms during which a white crosshair was displayed on a black background. Participants responded “same/different” via button press while the image was still on the screen. Before entering the scanner, participants completed a practice n-back task to ensure they understood task instructions.

### Epigenotyping

Epigenotyping details are provided in Supplemental Information. In brief, DNA isolated from peripheral blood mononuclear cells (PBMC) was subjected to bisulfite treatment which converts non-methylated cytosines to uracil and leaves methylated cytosines unmodified. We then amplified a 116-base pair region of *OXTR* containing CpG site -934^27^ (hg38, chr3: 8,769,121) using polymerase chain reaction, and assessed methylated cytosines via pyrosequencing. Reported epigenotypes are an average of three replicates.

To address concerns that changes in cell type frequency may confound methylation results^37^, we subsequently conducted a small pilot analysis showing that methylation of PBMC, CD3^+^ and CD4^+^ T cells, CD14^+^ monocytes, and CD19^+^ B cells at site -934 does not vary across these cell types. Detailed methodology and results are provided in Supplemental Information.

### fMRI analyses

Image acquisition and analysis details are provided in Supplemental Information. Statistical analysis was conducted using general linear models in FSL^38^. We first conducted a one-sample *T*-test to determine the main effect of attentional target, which identifies regions that are similarly activated to the task contrast (Attend Faces > Attend Houses) across individuals. This analysis was used to ensure that, when considered as a whole group, the present sample activated regions expected to be involved in face perception during the Attend Faces relative to the Attend Houses condition. Multiple comparisons correction was carried out using false discovery rate (FDR) *q* < 0.05 voxel significance level and spatial extent threshold (*k*) ≥ 10 contiguous voxels.

To determine the effect of *OXTR* methylation on task-specific activity, we added *OXTR* methylation to the model and tested for linear relationships between *OXTR* methylation and Attend Faces > Attend Houses neural response. *Z* statistic images were thresholded using clusters determined by *Z* > 2.3 and cluster-corrected significance threshold of *p* < 0.05. We tested for outliers and data points with undue influence by ensuring that the absolute value of mean- and median-standardized residuals of the full model were < 3 and Cook’s distance was *D* < 1^39^ for each individual. Four outliers were removed and the model was rerun at the whole-brain level. Methylation (*t* = 1.35, *p* = 0.257) and overall activation (*t* = 0.49, *p* = 0.659) did not significantly differ among those individuals identified as outliers and those retained in the analysis.

For all models, diagnostics performed in R^40^ determined that assumptions of normality of residuals, linearity, and heteroscedasticity were met after the removal of outliers and influential points. To illustrate significant effects, clusters that survived correction were registered to subject space and mean *Z*-statistic values were extracted for each participant from these clusters and plotted against their *OXTR* methylation value.

### Functional connectivity analysis

We then conducted a psychophysiological interaction (PPI) analysis^41^ to examine the effect of *OXTR* methylation on task-specific DLPFC functional connectivity. For the seed, we created a 10 mm sphere around the peak coordinates from DLPFC identified by Herrington and colleagues to differentiate ASD and control individuals during selective social attention^12^. A group-level analysis testing for linear relationships between *OXTR* methylation and Attend Faces > Attend Houses DLPFC connectivity proceeded exactly as described for the fMRI analysis. Four unique outliers were removed. Methylation (*t* = −0.58, *p* = 0.596) and overall activation (*t* = 1.12, *p* = 0.343) did not significantly differ among those individuals identified as outliers and those retained in the analysis.

### Behavioral performance analysis

To determine whether activation or connectivity during selective social attention impacted task performance, we ran logistic regression models^42^ in R predicting proportion of items correct from mean *Z*-statistic values extracted from each significant cluster identified in the fMRI analysis, the DLPFC connectivity network, and the interaction between each cluster and the connectivity network. Methylation was included as a nuisance regressor in all models. Identified outliers and influential points were removed from each model. Significant interactions were visualized using the effects package^43^ in R.

To determine whether variance in the activation or connectivity patterns identified in the methylation covariate analysis could be accounted for by task performance, we re-ran the fMRI and PPI analyses as described above with task performance included as an additional covariate.

### Social behavioral phenotype analysis

The social salience hypothesis of oxytocin posits that individual difference factors contribute to variable responses during social tasks. We therefore tested a secondary, exploratory hypothesis that traits associated with the social behavioral phenotype moderate the relationship between *OXTR* methylation and neural response during selective social attention in our healthy adult sample. To quantify the occurrence of autistic and social anxiety traits, participants completed the Autism Quotient Questionnaire (AQ)^44^, a fifty-item self-report measure that assesses traits and behaviors associated with autism, and the Social Interaction Anxiety Scale (SIAS)^45^, a twenty-item self-report measure that assesses anxiety experienced during social interaction.

To test the relationship between AQ score, SIAS score, *OXTR* methylation, and neural activation during selective social attention, we included each mean-centered measure (AQ, SIAS, *OXTR* methylation) and all interaction terms (AQ × SIAS, AQ × *OXTR* methylation, SIAS × *OXTR* methylation, AQ × SIAS × *OXTR* methylation) as regressors in a whole-brain model, and computed contrasts testing for linear relationships between each regressor and Attend Faces > Attend Houses activation. Two participants did not complete the questionnaires. Three outliers were removed and the model was rerun at the whole-brain level. Methylation (*t* = 1.39, *p* = 0.272), overall activation (*t* = −0.57, *p* = 0.625), AQ score (*t* = 1.54, *p* = 0.225), and SIAS score (*t* = 2.21, *p* = 0.083) did not significantly differ among those individuals identified as outliers and those retained in the analysis.

## Results

### Selectively attending to faces activates the face perception network

Despite the fact that faces are always present in the stimulus, we replicate previous work^34,46^ showing participants activate regions of the face perception network^47,48^ to a greater extent when selectively attending to faces (Fig. 1, Supplementary Table 2).

### Individuals with higher *OXTR* methylation show greater recruitment of regions of the attentional control network when selectively attending to social information

**Fig. 2:**
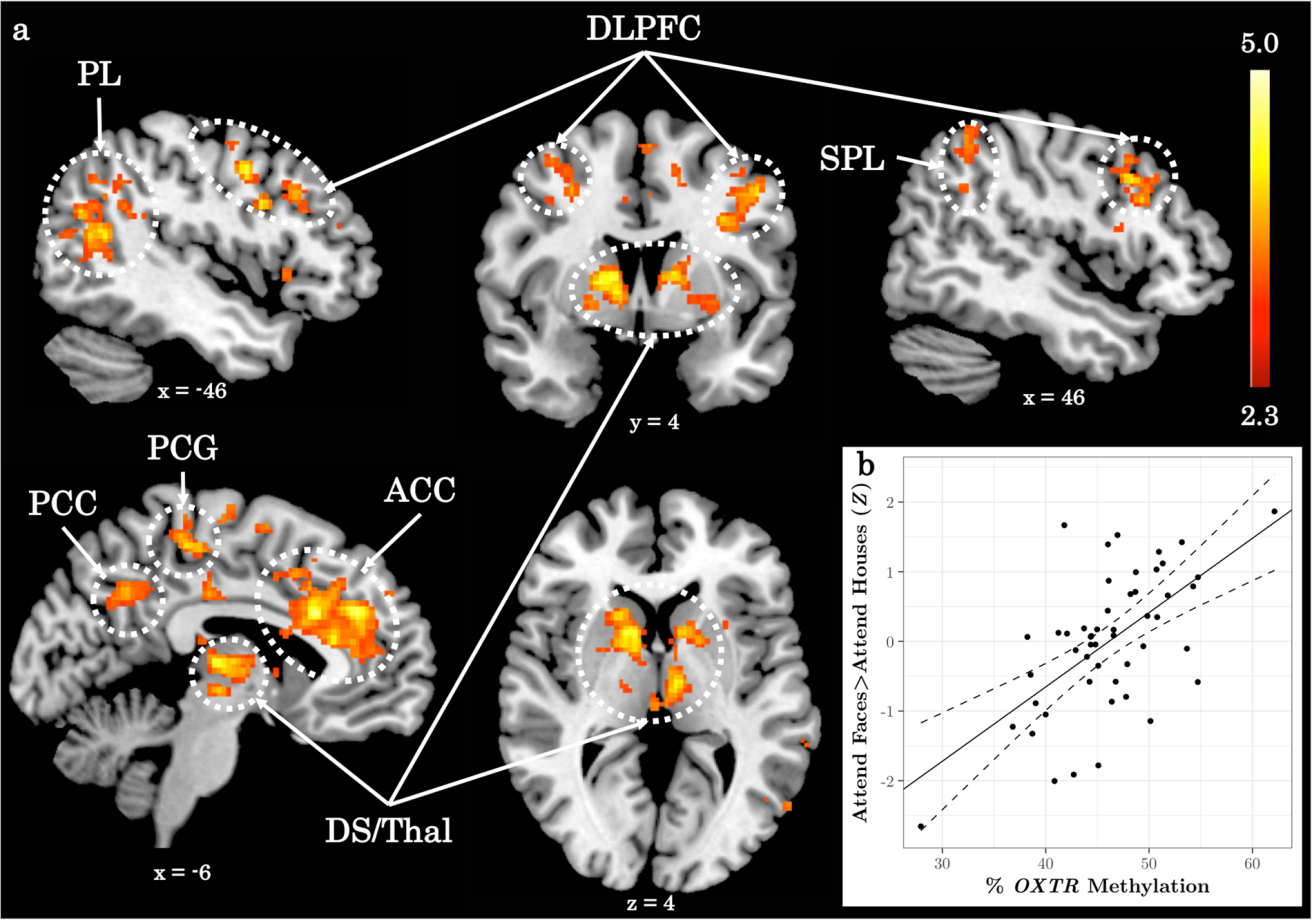
Individuals with higher *OXTR* methylation show greater recruitment of regions of the attentional control network when selectively attending to social information. **a** *Z* statistic map of regions showing a significant negative relationship between *OXTR* methylation and Attend Houses > Attend Faces BOLD activity, FWE cluster-corrected at *Z* > 2.3. Images are depicted in MNI space and radiological orientation. **b** Mean *Z* statistic values are plotted against percent *OXTR* methylation for each participant (*n* = 50). Solid line depicts the best-fit line; dashed lines depict 95% confidence interval. PL parietal lobule, DLPFC dorsolateral prefrontal cortex, SPL superior parietal lobule, PCC posterior cingulate cortex, PCG precentral gyrus, ACC anterior cingulate cortex, DS dorsal striatum, Thal thalamus

*OXTR* methylation ranged from 27.92 to 62.13% (*M* = 45.85%, SD = 5.69%). Methylation between males (*M* = 44.82%) and females (*M* = 47.25%) did not significantly differ (*t* = −1.57, *p* = 0.122). When testing for individual differences in the Attend Faces > Attend Houses contrast as a function of *OXTR* methylation, we find a significant positive association between *OXTR* methylation and regions of the attentional control network^2,7^, including bilateral DLPFC and bilateral parietal lobule (Fig. 2, Supplementary Table 3).

### Individuals with higher *OXTR* methylation exhibit decreased functional connectivity between DLPFC and regions of the salience network during selective attention to social information

**Fig. 3:**
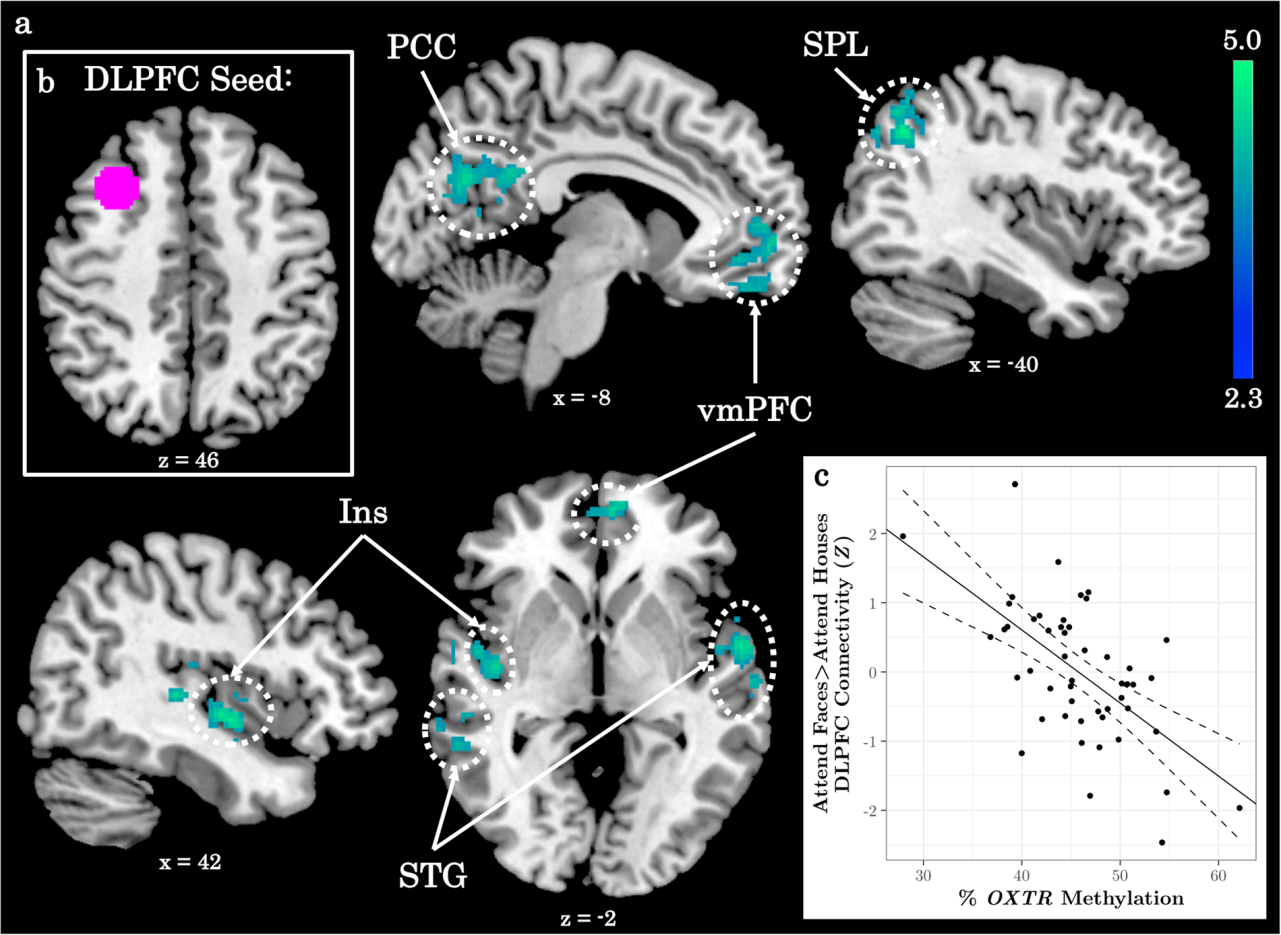
Individuals with higher *OXTR* methylation exhibit decreased functional connectivity between DLPFC and regions of the salience network during selective attention to social information. **a** *Z* statistic map of regions showing a significant negative relationship between *OXTR* methylation and right dorsolateral prefrontal cortex (DLPFC) connectivity, FWE cluster-corrected at *Z* > 2.3. Images are depicted in MNI space and radiological orientation. **b** DLPFC seed region. **c** Mean *Z* statistic values are plotted against percent *OXTR* methylation for each participant (*n* = 50). Solid line depicts the best-fit line; dashed lines depict 95% confidence interval. DLPFC dorsolateral prefrontal cortex, PCC posterior cingulate cortex, SPL superior parietal lobule, VMPFC ventromedial prefrontal cortex, Ins insular cortex, STG superior temporal gyrus

We ran a seed-based functional connectivity analysis to determine whether right DLPFC—a region previously shown to discriminate between ASD and control participants during selective social attention^12^—showed task-specific coupling with any other regions as a function of *OXTR* methylation. This analysis revealed a significant negative relationship between *OXTR* methylation and Attend Faces > Attend Houses DLPFC connectivity within regions of the salience network^3,7^, including right insula and bilateral superior temporal gyrus (Fig. 3, Supplementary Table 4).

### DLPFC activation and functional connectivity during selective social attention interact to impact task performance

Unlike many social-cognitive tasks in which healthy adults perform at ceiling, the selective social attention task is capable of generating significant variability in performance. Task accuracy ranged from 9.26 to 87.04% (*M* *=* 55.21%, SD *=* 21.78%). Accuracy did not significantly vary across Attend Faces (*M* *=* 53.60%) and Attend Houses (*M* = 56.82%) conditions (*t* = −0.75, *p* = 0.453). The performance of males (*M* = 55.64%) and females (*M* = 54.63%) did not significantly differ (*t* = 0.16, *p* = 0.870).

**Fig. 4:**
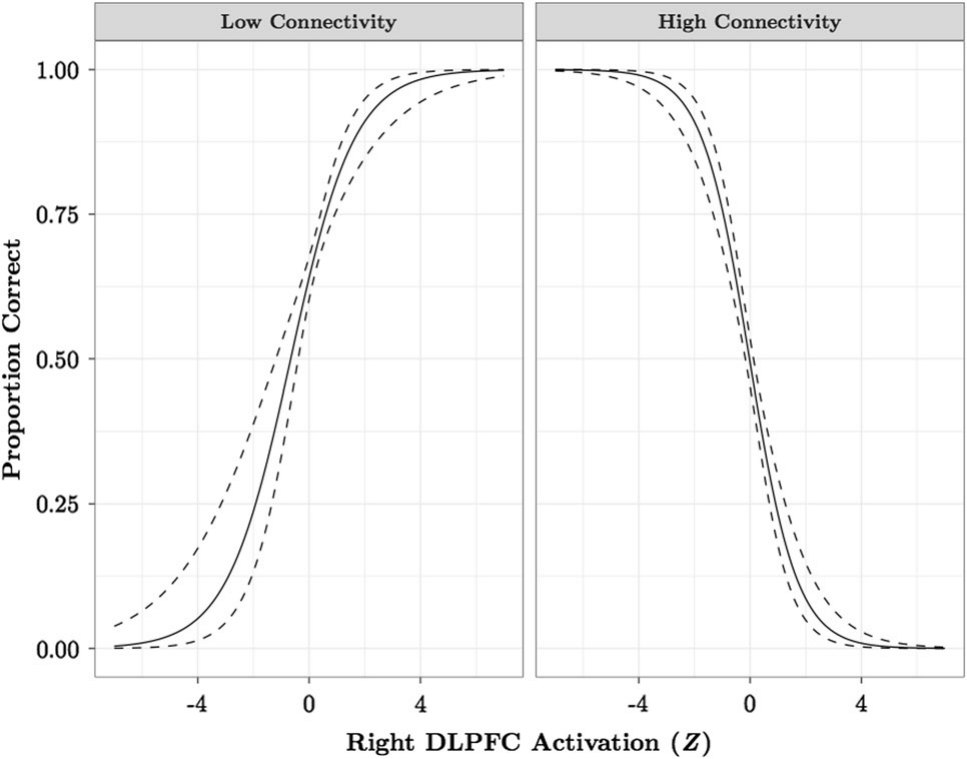
DLPFC activation and functional connectivity during selective social attention interact to impact task performance. Results of the logistic regression model predicting total proportion correct as a function of the interaction between right DLPFC activation and functional connectivity. To illustrate the interaction, we plot the relationship between DLPFC activation and +/−2 SD DLPFC connectivity. DLPFC dorsolateral prefrontal cortex

We ran logistic regression analyses to determine how activation and connectivity that vary as a function of *OXTR* methylation impact task performance. We find that the DLPFC functional connectivity network interacts with each region identified in the Attend Faces > Attend Houses analysis to predict task performance. The interaction is such that task performance for individuals with low connectivity improves with greater recruitment of attentional control regions, whereas individuals with high connectivity show poorer task performance with increased activation of these regions during selective social attention (Fig. 4; Supplementary Table 5).

Including task performance as an additional covariate did not appreciably change the pattern of activation identified in the fMRI or the PPI analyses; 89.09% of significant voxels for the fMRI analysis and 98.42% of significant voxels for the PPI analysis overlap with those identified in the respective analyses with only *OXTR* methylation included as a covariate (Supplementary Figure 1).

### Unique components of the social behavioral phenotype differentially impact the relationship between *OXTR* methylation and neural response during selective social attention

We assessed self-reported traits associated with autism and social anxiety in our sample of healthy adults to explore the hypothesis that individual difference factors moderate the relationship between *OXTR* methylation and neural response during selective social attention. AQ scores ranged from 7 to 32 (*M* = 17.13, SD = 5.48, skew = 0.52, kurtosis = 0.16), with males scoring significantly higher (*M* = 18.66) than females (*M* = 15.22), (*t* = 2.34, *p* = 0.023). SIAS scores ranged from 1 to 61 (*M* = 21.29, SD = 14.71, skew = 1.00, kurtosis = 0.17). Males (*M* = 22.07) and females (*M* = 20.30) did not significantly differ on SIAS score (*t* = 0.43, *p* = 0.672). AQ and SIAS scores were significantly positively correlated (*r* = 0.50, *p* < 0.0001).

**Fig. 5:**
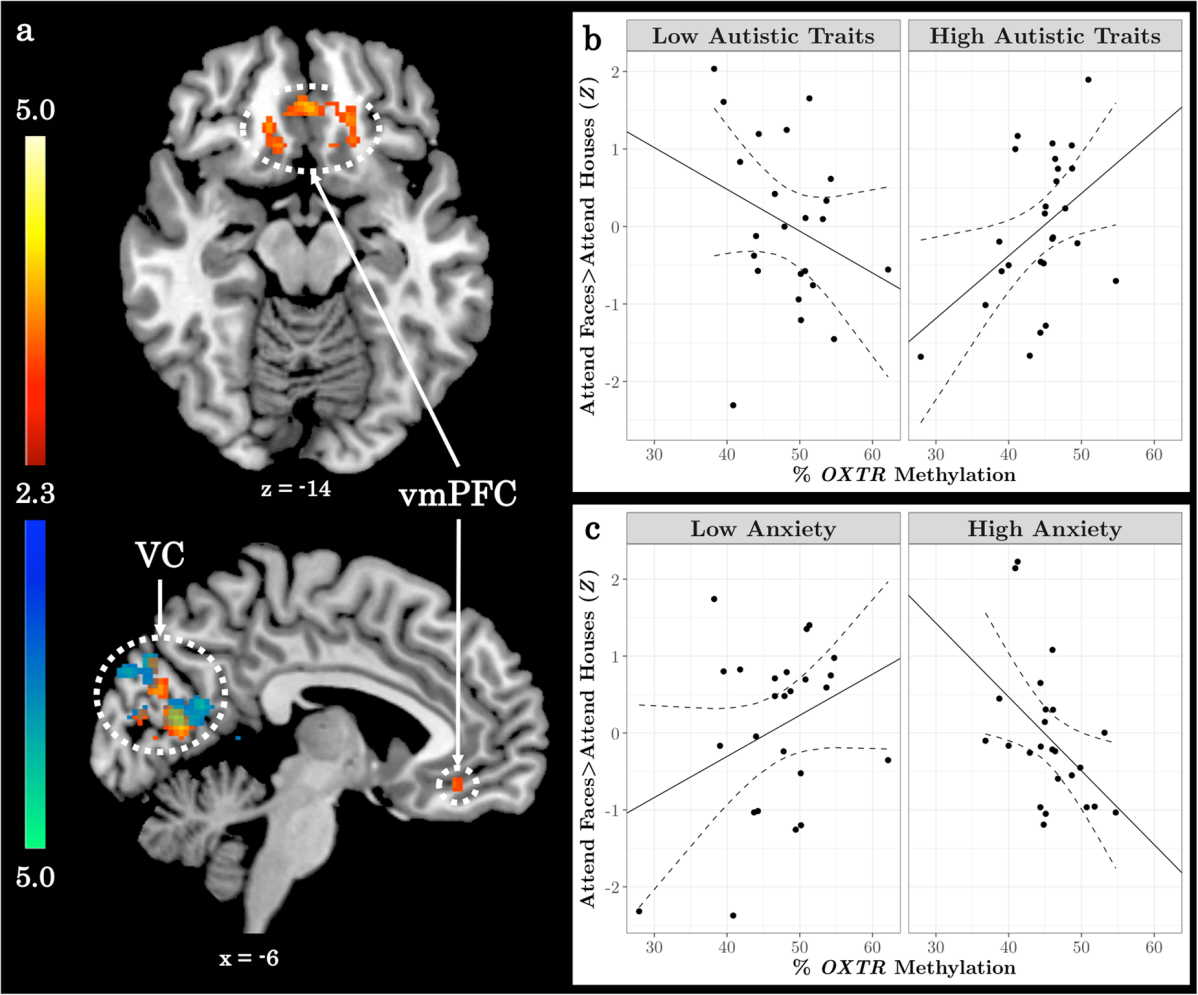
Unique components of the social behavioral phenotype differentially impact the relationship between *OXTR* methylation and neural response during selective social attention. **a** *Z* statistic map of regions showing a significant positive interaction between *OXTR* methylation and AQ score (hot colors), and a significant negative interaction between *OXTR* methylation and SIAS score (cool colors), FWE cluster-corrected at *Z* > 2.3. Images are depicted in MNI space and radiological orientation. **b**, **c** Mean *Z* statistic values are plotted against percent *OXTR* methylation for each participant (*n* = 49). To illustrate the interaction, (**b**) Autism Quotient (AQ) and (**c**) Social Interaction Anxiety Scale (SIAS) scores are plotted by median split. Solid lines depict the best-fit line; dashed lines depict 95% confidence interval. VC visual cortex, vmPFC ventromedial prefrontal cortex

An analysis assessing relationships between neural activation, *OXTR* methylation, AQ, and SIAS scores revealed a significant positive relationship between the AQ × *OXTR* methylation interaction within visual cortex and ventromedial prefrontal cortex, and a significant negative relationship between the SIAS × *OXTR* methylation interaction on activation within visual cortex (Fig. 5, Supplementary Table 6).

## Discussion

We show for the first time that individual variability in the endogenous oxytocinergic system is associated with differential response within neural networks responsible for guiding attention to social information. These data suggest that *OXTR* methylation reflects a decrease in the extent to which social information automatically captures attention, and offer a mechanistic explanation to a growing literature associating epigenetic modification to *OXTR* with social behavior and disorder^28,29,49–52^.

The brain regions that emerge in the present study largely overlap with key nodes of the attentional control and salience networks^1–5,7^. We find individuals with higher *OXTR* methylation display increased activation within anterior cingulate, parietal lobule, putamen/thalamus, and DLPFC. Herrington and colleagues previously showed that individuals with ASD recruit these same regions to a greater extent than typically developing controls when performing this task^12^. They concluded that individuals with ASD over-recruit these attentional control regions, particularly right DLPFC, to compensate for decreased face processing capabilities. We interpret greater activation in these same regions among individuals with higher *OXTR* methylation to indicate diminished intrinsic salience of social information for those with presumed decreased access to endogenous oxytocin.

We also demonstrate that higher *OXTR* methylation is associated with decreased functional coupling between regions of the salience and attentional control networks. We selected right DLPFC as the seed region in our functional connectivity analysis because it was previously shown to maximally differentiate between ASD and healthy controls in this task^12^. However, a larger literature suggests that this region links the dual attention networks and is particularly important for switching between networks^5,7,53^. Decreased functional coupling between DLPFC and salience regions might therefore reflect aberrant inter-regulation between attention networks for those with increased *OXTR* methylation during selective social attention.

Optimal attentional resource allocation requires a balance between both attention networks and cannot be attributed to either system in isolation^5^. We find the activation within and connectivity between attention networks that vary as a function of *OXTR* methylation interact to predict task performance. Improved performance for individuals with low connectivity/high activation might reflect a hypoactive or inefficient social detection system that benefits from increased effort to attend to the target stimulus^54^, whereas poor performance for individuals with high connectivity/high activation might indicate a failure to suppress hyperactive orienting systems that are typically deactivated when goal-directed attention regions like DLPFC are activated^7,55^. These results suggest that an imbalance between attention systems might be established by variability in the endogenous oxytocinergic system, perhaps leading to hypo- or hyper-attentional biases to social information. That the fMRI and PPI results do not appreciably change when task performance is included as a covariate further suggests that the interaction between these attentional systems is most important for predicting task performance, rather than functional activation or connectivity alone.

The social salience hypothesis of oxytocin emphasizes the need to account for individual difference factors that might drive differential response to oxytocin^21–23^, such as psychiatric disorders associated with hypo/hyper social-attentional biases^9–11,56^. In an exploratory analysis, we tested the hypothesis that even subclinical traits associated with social disorder would moderate the relationship between *OXTR* methylation and neural response during selective social attention. We find that, despite a significant correlation between measures of autistic and social anxiety traits, these unique aspects of the social behavioral phenotype show divergent relationships between *OXTR* methylation and neural activation within visual cortex. Visual cortex, which ultimately resolves competition among stimuli for neural representation, is activated with increased processing of visual information. A large body of literature suggests visual cortex activity can also be modulated by regions of the attentional control network^5,55,57^. Therefore, individuals with high autistic traits or low social anxiety might show increased visual cortex activation with increased *OXTR* methylation, which our data suggest is associated with increased attentional-control resources, because those with diminished social-attentional bias require upregulation of social information processing via attentional-control mechanisms. Conversely, individuals with low autistic traits or with higher social anxiety may show increased visual cortex activation with lower *OXTR* methylation because their increased social-attentional bias leads to enhanced social information processing without the need for additional attentional mechanisms.

### Avenues for future research

The present study excluded clinical populations, therefore we are limited in our ability to discuss the full trait continuum. Future work should probe these relationships in samples that include both clinical and nonclinical populations while considering neurobiological and phenotypic measures on a continuum. To understand the generalizability of these results, future research must also investigate these relationships across a wider range of age, racial, and ethnic groups. Previous work has demonstrated a similar relationship between methylation at *OXTR* site -934 and neural activity across racial groups^28^, but this area of research needs further study.

While our results are correlational in nature, by taking a candidate site approach we are able to build upon previous research demonstrating the functional relevance of site -934. Gregory, Connelly et al. assayed multiple CpG sites within two CpG islands on *OXTR* and found that only site -934 methylation was both significantly negatively associated with gene expression in human cortex, suggesting a functional role in regulating gene transcription, and highly variable and elevated in both brain and blood of individuals with autism, suggesting this marker is indicative of phenotypic variability and conserved across tissues^27^. This latter point is particularly important because methylation plays a role in cell-type differentiation causing methylation patterns to vary across tissues. Epigenetic association studies, particularly those investigating age- and disease-related associations, have therefore recently come under criticism because cell type frequency also varies across ages and disease states. Such variability in cellular composition of a tissue may confound or even drive the differences in methylation patterns seen across age groups or disease states. This may be particularly problematic for epigenome wide association studies that often show very small (<5%) variability in methylation and therefore must account for all sources of variation^37,58^. Methylation at site -934 shows wide variation (27.92–62.13% in the present sample) and our small pilot study suggests that individual differences in methylation at our tested site are independent of cellular heterogeneity. This result warrants replication in a larger sample and across additional cell types. Future research taking advantage of recent advances in in vitro technology that enable the manipulation of methylation state^59^ is needed to establish whether a causal link exists between *OXTR* methylation and variability of the social behavioral phenotype and supporting neural systems.

## Conclusion

Together, our results demonstrate that epigenetic variability in the oxytocinergic system is associated with variable neural response within attention networks during selective social attention, which is differentially associated with social information processing across social behavioral phenotypes. Despite high comorbidity among autistic and anxious phenotypes^60^, we demonstrate that these are separable aspects of the social behavioral phenotype and add to literature suggesting these phenotypes reflect distinct differences in the oxytocinergic system^27,49^. That these results emerge within a nonclinical sample highlights the importance of considering “healthy” variability, which may be of particular importance for biomarker or treatment studies that traditionally dichotomize groups by diagnosis.

## Electronic supplementary material

Supplemental Information
