# Supplementary material for: Epigenetic regulation of the oxytocin receptor is associated with neural response during selective social attention
Source: Transl Psychiatry. 2018 Jun 15;8:116. doi: 10.1038/s41398-018-0159-x (PMC6003910; doi:10.1038/s41398-018-0159-x)
Supplement: Supplementary file 1 — Supplemental Information [file 41398_2018_159_MOESM1_ESM.pdf]

Supplemental Information

Materials and Methods

Epigenotyping. A professional phlebotomist collected 8 milliliters of blood from each participant in mononuclear cell separation tubes (BD Vacutainer CPT with sodium citrate, BD Biosciences, Franklin Lanes, NJ). The blood draw was completed directly before or after the fMRI component, counterbalanced across participants. We immediately spun blood samples at 1800 RCF for 30 min to separate mononuclear cells per product protocol, then lysed the mononuclear cells and extracted DNA using reagents supplied in the Gentra Puregene Blood Kit (Qiagen, Valencia, CA). Two hundred nanograms of DNA was subject to bisulfite treatment (Kit MECOV50, Invitrogen, Carlsbad, CA), which converts non-methylated cytosines to uracil for downstream detection of methylated cytosines by sequencing.

We amplified a 116-base pair region of *OXTR* containing CpG Site -934[1] (hg38, chr3: 8 769 121) via polymerase chain reaction (PCR) using 12 nanograms of bisulfite-converted DNA, 0.2 µM primers TSL101F (5′-TTGAGTTTTGGATTTAGATAATTAAGGATT-3′) and TSL101R (5′-biotin-AATAAAATACCTCCCACTCCTTATTCCTAA-3′), and reagents supplied in the Pyromark PCR kit (Qiagen, Valencia, CA). Underlined nucleotides in primer set indicate the insertion of an A or C nucleotide at a variable position (C/T) due to a CpG site within the primer. Samples were amplified in triplicate on three identical PCR machines (S1000 Thermal Cycler, Biorad, Hercules, CA) with the following cycling conditions [Step 1: (95°C/15 min)/1 cycle, Step 2: (94°C/30 s, 56°C/30 s, 72°C/30 s)/50 cycles, Step 3: (72°C/10 min)/1 cycle, Step 4: 4°C hold]. Pyrosequencing was performed using primer TSL101S (5′-AGAAGTTATTTTATAATTTTT-3′) on a Pyromark Q24 using PyroMark Gold Q24 Reagents (Qiagen, Valencia, CA). We included methylation controls at 0, 25, 50, 75, and 100%

methylated and find significant correlation ($r > 0.99$, $p < .0001$) between experimental and expected values. Reported epigenotypes are an average of three replicates. On average, replicates deviated from the mean ±1.47%.

Cell Type Isolation. To determine whether methylation at the targeted CpG site varies by cell type, we conducted a small pilot analysis testing methylation across Peripheral blood mononuclear cells (PBMC), $CD3^+$ and $CD4^+$ T cells, $CD14^+$ monocytes, and $CD19^+$ B cells. Three Caucasian participants provided a total of 40 mL of blood collected in 5 mononuclear cell separation tubes (BD Vacutainer CPT with sodium citrate, BD Biosciences, Franklin Lanes, NJ). PBMC isolation proceeded exactly as described above, except prior to cell lysis individual cell types were isolated using reagents supplied in the EasySep Human CD3, CD4, CD14 and CD19 Positive Selection Kits (StemCell, Vancouver, Canada) per product protocol. Epigenotyping was performed as described above. On average, replicates deviated from the mean ±2.42%. We performed a repeated measures ANOVA with cell type (PBMC, $CD3^+$, $CD4^+$, $CD14^+$, $CD19^+$) as within-subjects' factor to statistically examine the effects of cell type on methylation at CpG site -934. Model diagnostics determined that assumptions of normality and homogeneity of variances were met.

Image acquisition and preprocessing. Scanning was performed at the University of Virginia on a Siemens 3 Tesla MAGNETOM Trio high-speed imaging device equipped with a 12-channel head-coil. High-resolution T1-weighted anatomical images were first acquired using Siemens' magnetization-prepared rapid-acquired gradient echo (MPRAGE) pulse sequence with the following specifications: echo time (TE) = 2.53 ms; repetition time (TR) = 1900 ms; flip angle (FA) = 9°; field-of-view (FOV) = 250 mm; image matrix = 256 mm × 256 mm; slice thickness = 1 mm; 176 slices. Whole-brain functional images were then acquired using a

$T2^*$ weighted echo planar imaging (EPI) sequence sensitive to blood oxygenation level dependent (BOLD) contrast with the following specifications: TE = 40 ms; TR = 2000 ms; FA = 90°; FOV = 192 mm; image matrix = 64 mm x 64 mm; slice thickness = 3.5 mm; slice gap = 22%; 260 volumes of 28 slices co-planar with the anterior and posterior commissures. Stimuli were presented with Psychophysics Toolbox[2] for MATLAB using an LCD AVOTEC projector onto a screen located behind the subject's head and viewed through an integrated head-coil mirror.

Data preprocessing was carried out using FEAT (FMRI Expert Analysis Tool) Version 6.00, part of FSL (FMRIB Software Library).[3] Motion was assessed by center of mass measurements (BXH/XCEDE Tools, version 1.8.16, Bioinformatics Information Research Network) to ensure that no participants had greater than a 2-mm deviation in the x-, y-, or z-dimensions. The following pre-statistics processing was applied: motion correction using MCFLIRT;[4] slice timing correction using Fourier-space time-series phase-shifting; non-brain removal using BET;[5] spatial smoothing using a Gaussian kernel of 5.0 mm full width at half maximum; grand-mean intensity normalization of the entire 4D dataset by a single multiplicative factor; high-pass temporal filtering (Gaussian-weighted least-squares straight line fitting, with sigma = 50.0 s). Additionally, each functional volume was registered to the participant's high resolution anatomical image, and then to FSL's standard Montreal Neurologic Institute (MNI 152, T1 2mm) template brain using FSL's linear registration tool (FLIRT).[4] Registration from high resolution structural to standard space was then further refined using FSL's nonlinear registration, FNIRT.[6,7]

fMRI analyses. Imaging analysis was conducted using FEAT (FMRI Expert Analysis Tool) Version 6.00, part of FSL.[3] At the subject level, time-series statistical analysis was carried

out using FSL's improved linear model (FILM) with local autocorrelation correction.[8] Regressors for each condition (Attend Faces, Attend Houses) were modeled by convolving the time course with a double-gamma hemodynamic response function (HRF), adding a temporal derivative, and applying temporal filtering. An Attend Faces>Attend Houses contrast was computed and the contrast of parameter estimates (COPE) from this analyses for each individual was carried forward to higher-level analysis. Group-level analyses were conducted at the whole-brain level using FSL's local analysis of mixed effects (FLAME) stage 1.[9–11]

Functional connectivity analyses. First-level psychophysiological interaction (PPI) analysis[12] was carried out in FEAT, with time-series statistical analysis carried out using FILM with local autocorrelation correction. Regressors included: the psychological variable: Attend Faces – Attend Houses (convolved with a double-gamma HRF; temporal derivative added; temporal filtering applied); the physiological variable: mean DLPFC seed-region time-series; the PPI variable: interaction term between the psychological regressor (zero-centered about min and max values) and the physiological regressor (mean-centered); and the Attend Faces + Attend Houses time course (convolved with a double-gamma HRF; temporal derivative added; temporal filtering applied) to account for shared variance between conditions. Higher-level analysis proceeded exactly as described for the fMRI analysis except each participant's PPI COPE replaced the Attend Faces>Attend Houses activation COPE.

Results

Methylation across cell types. A repeated measures ANOVA with cell type (PBMC, $CD3^+$, $CD4^+$, $CD14^+$, $CD19^+$) as within-subjects' factor revealed no significant effect of cell type on *OXTR* methylation at CpG site -934 ($F(4,8) = 2.81$, $p = .100$). Methylation means and standard deviations for each cell type are listed in Supplementary Table 1.

**Cell Type**	***M***	***SD***
PBMC	48.00	3.90
$CD3^+$	48.88	4.53
$CD4^+$	47.83	3.16
$CD14^+$	40.87	7.33
$CD19^+$	44.42	1.92

Supplementary Table 1. Percent methylation values by cell type. *M*, mean; *SD*, standard deviation; PBMC, peripheral blood mononuclear cells; $CD3^+$, T cells; $CD4^+$, T cells; $CD14^+$, monocytes; $CD19^+$, B cells.

Contrast	Anatomical region	Hem	x	y	z	*Z*	k
	Cuneus	B	2	-84	20	6.62	2700
	Rostromedial prefrontal cortex	B	-4	54	26	6.32	2796
	Posterior superior temporal cortex	R	60	-40	0	6.21	2214
	Ventromedial prefrontal cortex	B	-2	38	-24	5.71	610
	Orbitofrontal cortex	L	-46	28	-12	5.70	2535
	Orbitofrontal cortex	R	48	34	-6	5.11	613
	Amygdala	R	18	-6	-20	5.03	178
	Lateral occipital cortex	L	-52	-54	54	4.60	316
	Posterior superior temporal cortex	L	-44	-64	18	4.49	792
	Caudate	L	-10	12	2	4.30	167
	Fusiform gyrus	L	-40	-46	-20	4.26	66
	Anterior cingulate cortex	R	6	28	-6	4.13	136
	Dorsal anterior cingulate cortex	L	-2	-16	38	4.10	101
	Lateral occipital cortex	R	52	-64	42	4.04	70
	Insular cortex	L	40	-2	-16	3.90	15
Attend Faces > Attend Houses	Postcentral gyrus	L	-16	-38	76	3.83	23
	Caudate	R	12	0	10	3.56	77
	Supramarginal gyrus	L	-62	-40	40	3.46	23
	Precentral gyrus	L	-20	-16	68	3.44	16
	Fusiform gyrus	R	46	-44	-20	3.43	11
	Precentral gyrus	R	24	-18	70	3.42	29
	Insular cortex	L	-58	-8	10	3.40	12
	Precentral gyrus	R	28	-26	62	3.38	20
	Supramarginal gyrus	L	-64	-50	24	3.31	16
	Orbitofrontal cortex	R	32	16	-24	3.24	13
	Insular cortex	L	-40	-8	-16	3.18	12
	Insular cortex	R	58	-20	12	3.11	17
	Middle temporal gyrus	L	-52	-4	-18	3.07	25
	Postcentral gyrus	L	-38	-30	66	3.05	14
	Dorsolateral prefrontal cortex	R	60	22	18	3.02	13
	Supramarginal gyrus	L	-54	-44	28	2.92	14
	Lateral occipital cortex	B	-28	-36	-20	10.3	27908
	Middle frontal gyrus	R	26	4	44	6.45	1449
	Middle frontal gyrus	L	-22	6	44	6.07	1031
	Thalamus	L	-18	-30	4	5.24	150
	Precentral gyrus	R	42	2	20	4.87	307
	Insular cortex	R	32	20	0	4.21	188
	Dorsolateral prefrontal cortex	R	48	44	20	4.20	299
	Precentral gyrus	L	-50	2	22	4.08	190
Attend Faces < Attend Houses	Pallidum	R	20	-10	-2	3.88	48
	Pallidum	L	-18	-8	-2	3.80	69
	Anterior cingulate cortex	B	2	6	24	3.31	10
	Thalamus	R	12	-20	8	3.29	10
	Dorsolateral prefrontal cortex	R	44	58	2	3.11	11
	Dorsolateral prefrontal cortex	R	42	58	12	3.03	29
	Postcentral gyrus	R	58	-12	26	2.96	15
	Insular cortex	R	40	-20	24	2.90	12
	Brainstem	B	-2	-28	-16	2.89	12
	Precentral gyrus	R	16	-14	48	2.82	10

Supplementary Table 2. Local maxima statistics for task main effect analysis. Significant voxel threshold: FDR(q) < .05, k > 10. Hem, hemisphere; B, bilateral; L, left; R, right; x, y, z, coordinates of local maxima in MNI space; Z, maximum Z statistic; k, cluster extent.

**Anatomical region**	**Hem**	**x**	**y**	**z**	***Z***	**k**
Anterior cingulate cortex	B	-12	28	16	4.61	4155
Dorsal striatum/Thalamus	R	10	8	10	4.26	587
Dorsolateral prefrontal cortex	L	-48	2	42	4.22	272
Dorsolateral prefrontal cortex	R	42	14	36	4.18	594
Superior parietal lobule	L	-58	-34	40	4.13	453
Dorsal striatum/Thalamus	L	-14	0	10	4.06	909
Posterior cingulate cortex	B	4	-12	36	3.99	832
Parietal lobule	L	-48	-58	18	3.81	771
Superior parietal lobule	R	54	-50	42	3.79	615
Precentral gyrus	B	-6	-32	56	3.52	782

Supplementary Table 3. Local maxima statistics for regions showing a significant positive relationship between *OXTR* methylation and Attend Faces > Attend Houses BOLD activity. Significant cluster threshold: $Z > 2.3$. Hem, hemisphere; B, bilateral; L, left; R, right; x, y, z, coordinates of local maxima in MNI space; *Z*, maximum *Z* statistic; k, cluster extent.

Anatomical region	Hem	x	y	z	*Z*	k
Precuneus/Posterior cingulate cortex	B	2	-78	32	4.61	2101
Superior parietal lobule	L	-38	-66	40	4.45	429
Insular cortex	R	50	-10	-10	3.81	461
Superior temporal gyrus	L	-60	-2	-4	3.71	297
Ventromedial prefrontal cortex	B	-16	54	-12	3.46	610
Superior temporal gyrus	R	52	-26	14	3.41	338

Supplementary Table 4. Local maxima statistics for regions showing a significant negative relationship between *OXTR* methylation and dorsolateral prefrontal cortex connectivity. Significant cluster threshold: $Z > 2.3$. Hem, hemisphere; B, bilateral; L, left; R, right; x, y, z, coordinates of local maxima in MNI space; *Z*, maximum *Z* statistic; k, cluster extent.

Anatomical region	Hem	β	SE	P	DF
Anterior cingulate cortex	B	-0.20	0.04	< .0001	38
Dorsal striatum/Thalamus	R	-0.25	0.05	< .0001	37
Dorsolateral prefrontal cortex	L	-0.41	0.04	< .0001	41
Dorsolateral prefrontal cortex	R	-0.29	0.04	< .0001	39
Superior parietal lobule	L	-0.18	0.04	< .0001	38
Dorsal striatum/Thalamus	L	-0.28	0.05	< .0001	38
Posterior cingulate cortex	B	-0.17	0.05	.0005	39
Inferior parietal lobule	L	-0.18	0.05	.0003	39
Superior parietal lobule	R	-0.37	0.04	< .0001	38
Precentral gyrus	B	-0.26	0.04	< .0001	38

Supplementary Table 5. Results of logistic regression models predicting task accuracy from the interaction between each anatomical region and the DLPFC functional connectivity network. All models included % *OXTR* methylation as a nuisance regressor. Any identified outliers and influential points were removed. Hem, hemisphere; B, bilateral; L, left; R, right; β, interaction coefficient, SE, standard error; P, p-value; DF, degrees of freedom.

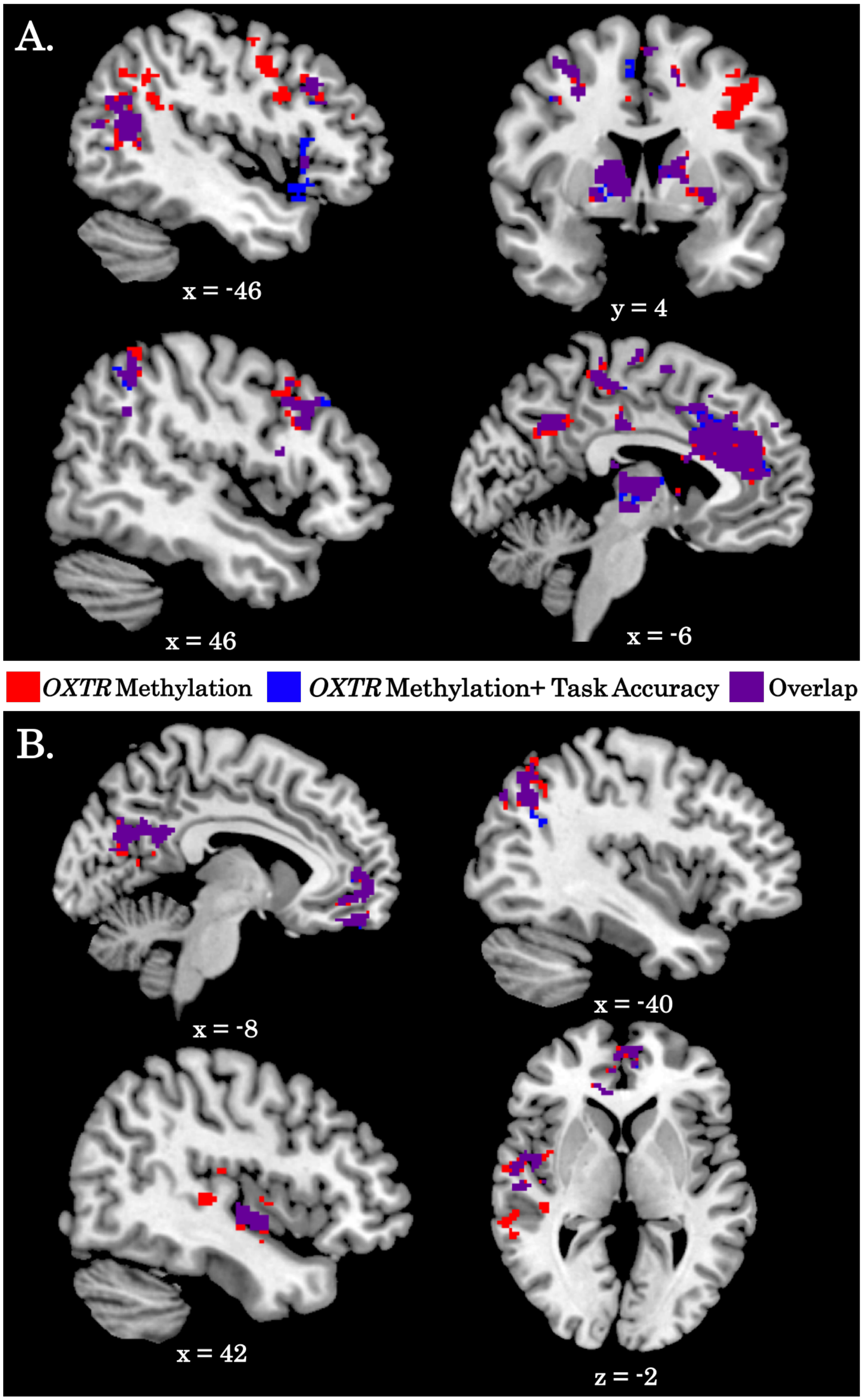

Supplementary Figure 1. Including task performance as a covariate does not impact (A) fMRI or (B) connectivity results. Comparison of activation identified in the methylation (red clusters) and methylation + task performance (blue clusters) covariate analyses. Purple voxels indicate those that overlap between these analyses. Coordinates are in MNI space.

Social Measure	Anatomical region	Hem	x	y	z	*Z*	k
AQ	Visual cortex	B	-12	-74	12	3.78	703
	Ventromedial prefrontal cortex	B	14	32	-12	3.35	311
SIAS	Visual cortex	B	-2	-72	18	3.95	921

Supplementary Table 6. Local maxima statistics for regions showing a significant interaction between *OXTR* methylation and components of the social behavioral phenotype. Significant cluster threshold: $Z > 2.3$. AQ, Autism Quotient; SIAS, Social Interaction Anxiety Scale; Hem, hemisphere; B, bilateral; x, y, z, coordinates of local maxima in MNI space; *Z*, maximum *Z* statistic; k, cluster extent.

References

1 Gregory SG, Connelly JJ, Towers AJ, Johnson J, Biscocho D, Markunas C a *et al.* Genomic and epigenetic evidence for oxytocin receptor deficiency in autism. *BMC Med* 2009; **7**: 62.

2 Brainard DH. The Psychophysics Toolbox. *Spat Vis* 1997; **10**: 443–446.

3 Smith SM, Jenkinson M, Woolrich MW, Beckmann CF, Behrens TEJ, Johansen-Berg H *et al.* Advances in functional and structural MR image analysis and implementation as FSL. *Neuroimage* 2004; **23**: S208-19.

4 Jenkinson M, Bannister P, Brady M, Smith S. Improved optimization for the robust and accurate linear registration and motion correction of brain images. *Neuroimage* 2002; **17**: 825–841.

5 Smith SM. Fast robust automated brain extraction. *Hum Brain Mapp* 2002; **17**: 143–155.

6 Andersson JLR, Jenkinson M, Smith S. Non-linear optimisation FMRIB Technial Report TR07JA1. Oxford, UK, 2007.

7 Andersson JLR, Jenkinson M, Smith S. Non-linear registration aka Spatial normalisation FMRIB Technial Report TR07JA2. Oxford, UK, 2007.

8 Woolrich MW, Ripley BD, Brady M, Smith SM. Temporal autocorrelation in univariate linear modeling of FMRI data. *Neuroimage* 2001; **14**: 1370–86.

9 Beckmann CF, Jenkinson M, Smith SM. General multilevel linear modeling for group analysis in FMRI. *Neuroimage* 2003; **20**: 1052–1063.

10 Woolrich MW, Behrens TEJ, Beckmann CF, Jenkinson M, Smith SM. Multilevel linear modelling for FMRI group analysis using Bayesian inference. *Neuroimage* 2004; **21**: 1732–1747.

11 Woolrich M. Robust group analysis using outlier inference. *Neuroimage* 2008; **41**: 286–301.

12 O'Reilly JX, Woolrich MW, Behrens TEJ, Smith SM, Johansen-Berg H. Tools of the trade: Psychophysiological interactions and functional connectivity. *Soc Cogn Affect Neurosci* 2012; **7**: 604–609.
